# An *Arabidopsis thaliana* (Ler) inbred line exhibiting stacked stem/inflorescences mainly due to the reduced AP1 expression

**DOI:** 10.1186/1753-6561-5-S7-P68

**Published:** 2011-09-13

**Authors:** Xiaoli Qi, Yao Jiang, Fang Tang, Shu-Tang Zhao, Meng-Zhu Lu

**Affiliations:** 1 College of Life Sciences, Jiamusi University, Jiamusi 154007, China; 2 State Key Lab of Forest Genetics and Tree Breeding, Chinese Academy of Forestry, Beijing 100091, China

## Background

Bolting is regarded as the initiation of reproduction stage in the growth and development of an Arabidopsis plant, when a set of floral integrator genes activate the expression of floral meristem identity genes *LFY* and *AP1* to initiate flowering transition[1,2] . However, how the expression of key genes, such as *AP1*, responds of diverse signals during flower development remains largely unknown. Here we have obtained an inbred line exhibiting an abnormal stem/inflorescence and flower development.

## Methods

The morphological variations were carefully observed visually or with a Zeiss SteREO Discovery V8 stereomicroscope. Hybridization of GeneChip arrays was done in an Affymetrix Hybridization Oven 640 following the manufacturer’s protocol (Affymetrix) and expression levels in seedlings and flower buds were calculated from Affymetrix intensity data. Real-time PCR was performed on an ABI 7500 Real-Time PCR System (Applied Biosystems, Shanghai, China) with SYBR GreenPCR Master Mix (Applied Biosystems) as the fluorescence source.

## Results

**Figure 1:**
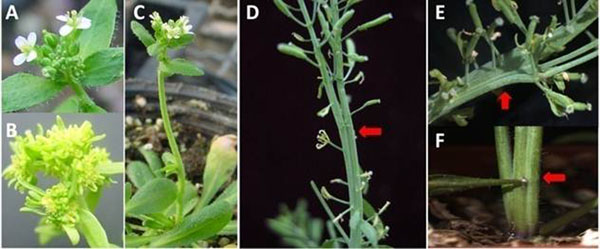
Phenotype of Wt and An inbred line.A, C: Wt; B, D-F: An inbred line)

The inbred line exhibits a flower phenotype similar to the *ap1*, such as homeotic conversion of sepals (first whorl) to leaf-like, petals often absent (second whorl) and complete flower-to-inflorescence conversions (Figure1, B). However flower meristem was replaced by emerging inflorescence meristems thus leading to a stacked stem/inflorescences before final flowering (Figure1, D). Position and degree of stacked stem/inflorescences are varied differently (Figure1, E and F).

**Figure 2:**
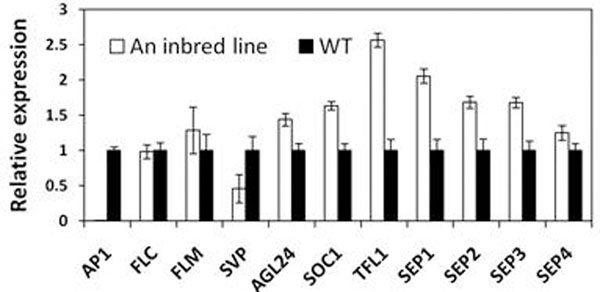
Expression of flowering regulatory genes in flower buds using qRT-PCR

Microarray and quantitative real-time PCR analysis revealed that the expression of *AP1* was significantly reduced, while the expression of its interacting genes *TERMINAL FLOWER 1* (*TFL1*), *OVEREXPRESSION OF CONSTANS(SOC1)*, *AGAMOUS-like 24* (*AGL24*), *SEPALLATA* (*SEP*) and upstream genes *FLOWERING LOCUS M* (*FLM*) were increased in flower buds (Figure2).

*AP1* sequence analysis showed that the promoter, coding sequences and intron splice sites of *AP1* genomic sequence in this inbred line were unchanged comparing to that in wildtype, suggesting the complexity in the regulation of AP1 in the line. Therefore these synthetic contributions caused the development of this unique phenotype. For instance, TFL1 was found to be highly expressed, and this gene can negatively regulate AP1 and specify inflorescence meristem identity leading to a delay of floral meristem formation[3]. On the other hand, the low levels of AP1 in flower buds cannot repress expression of *AGL24* and *SOC1*, which promote inflorescence fates rather than flower formation in the meristem and result in more abundant and longer inflorescences[4]. This expression variation of these genes is subjected to a threshold[4], leading to an ON/OFF expression pattern of the master regulatory gene(s) (like *AP1*) to specify a floral or a stem/inflorescence meristem.

## Conclusions

The inbred line identified in this study is phenotypically similar to *ap1* mutants with noticeable deviations. The abnormal stem/inflorescence of the inbred line was mainly caused by significant downregulation of *AP1*, but also is attributable to crosstalk among key genes like *TFL1*, *AGL24, SOC1*, etc. to control the transition of vegetative growth to the flowering phase. The inbred linemerits further molecular characterization to understand better the regulatory molecular network.
